# Image-based phenotyping of seed architectural traits and prediction of seed weight using machine learning models in soybean

**DOI:** 10.3389/fpls.2023.1206357

**Published:** 2023-09-12

**Authors:** Nguyen Trung Duc, Ayyagari Ramlal, Ambika Rajendran, Dhandapani Raju, S. K. Lal, Sudhir Kumar, Rabi Narayan Sahoo, Viswanathan Chinnusamy

**Affiliations:** ^1^ Division of Plant Physiology, Indian Council of Agricultural Research-Indian Agricultural Research Institute (ICAR-IARI), New Delhi, India; ^2^ Vietnam National University of Agriculture, Hanoi, Vietnam; ^3^ Division of Genetics, Indian Council of Agricultural Research-Indian Agricultural Research Institute (ICAR-IARI), New Delhi, India; ^4^ School of Biological Sciences, Universiti Sains Malaysia (USM), Georgetown, Penang, Malaysia; ^5^ Division of Agricultural Physics, Indian Council of Agricultural Research-Indian Agricultural Research Institute (ICAR-IARI), New Delhi, India

**Keywords:** soybean, RGB image, machine learning, hundred-seed weight prediction, superior donor identification

## Abstract

Among seed attributes, weight is one of the main factors determining the soybean harvest index. Recently, the focus of soybean breeding has shifted to improving seed size and weight for crop optimization in terms of seed and oil yield. With recent technological advancements, there is an increasing application of imaging sensors that provide simple, real-time, non-destructive, and inexpensive image data for rapid image-based prediction of seed traits in plant breeding programs. The present work is related to digital image analysis of seed traits for the prediction of hundred-seed weight (HSW) in soybean. The image-based seed architectural traits (i-traits) measured were area size (AS), perimeter length (PL), length (L), width (W), length-to-width ratio (LWR), intersection of length and width (IS), seed circularity (CS), and distance between IS and CG (DS). The phenotypic investigation revealed significant genetic variability among 164 soybean genotypes for both i-traits and manually measured seed weight. Seven popular machine learning (ML) algorithms, namely Simple Linear Regression (SLR), Multiple Linear Regression (MLR), Random Forest (RF), Support Vector Regression (SVR), LASSO Regression (LR), Ridge Regression (RR), and Elastic Net Regression (EN), were used to create models that can predict the weight of soybean seeds based on the image-based novel features derived from the Red-Green-Blue (RGB)/visual image. Among the models, random forest and multiple linear regression models that use multiple explanatory variables related to seed size traits (AS, L, W, and DS) were identified as the best models for predicting seed weight with the highest prediction accuracy (coefficient of determination, R^2=^0.98 and 0.94, respectively) and the lowest prediction error, i.e., root mean square error (RMSE) and mean absolute error (MAE). Finally, principal components analysis (PCA) and a hierarchical clustering approach were used to identify IC538070 as a superior genotype with a larger seed size and weight. The identified donors/traits can potentially be used in soybean improvement programs

## Background

Soybean (*Glycine max* (L.) Merr.) is an important oilseed and food crop consumed worldwide. The seeds are protein-rich (40%), thereby making them a key crop for global food and nutrition security (Rajendran and Lal, 2020; Rajendran et al., 2022). It provides a good amount of essential amino acids, macronutrients, micronutrients, and minerals (Garda et al., 2020). Approximately two-thirds of the world’s protein concentrate requirements for livestock feed and 25% of global edible oil consumption are met by soybeans. The top producers of soybean include the USA, Brazil, and Argentina (referred to as the “Big 3”), while in India it is grown in the Kharif season and mainly produced in Madhya Pradesh, Maharashtra, Rajasthan, Karnataka, Andhra Pradesh, and Chhattisgarh (Agarwal et al., 2013; Siamabele, 2021). Global soybean productivity has increased from 265,088,429 metric tons in 2010 to 333,671,692 metric tons in 2019, while in India it has increased from 12,736,000 metric tons in 2010 to 13,267,520 metric tons in 2019 (FAOSTAT, 2019). Also, about 10.5 million metric tons of soybean oil are being consumed globally (SoyStats, 2020). In turn, breeding efforts are required to improve the status of soybean productivity for economically important traits such as seed shape and weight (Kumar et al., 2023). Soybeans are widely used in various traditional and modern preparations, such as soy paste, soy milk, miso, tofu, and natto, in addition to soybean meal (Medic et al., 2014). Soybean harbors a wide variety of compounds with therapeutic roles, such as storage proteins used to treat hypocholesterolemia and chronic kidney diseases. In addition, trypsin inhibitors and lectins have anti-cancer properties, and soy compounds and iso-flavonoids can act against angiotensin-converting enzymes along with other constituents and provide scope for crop improvement (Medic et al., 2014; Ramlal et al., 2022a; Ramlal et al., 2022b; Ramlal et al., 2022c; Ramlal et al., 2023a). Soybean is a major legume consumed by both humans and livestock (Rajendran et al., 2023). Another important aspect of soybean, when genetically modified, is its herbicide tolerance, which has revolutionized production and reduced the use of pesticides (Nandula, 2019). It is also used in sealing agents and adhesives, biofuel production, pharmaceuticals, and the food industry (Lee et al., 2007). Soybean is also involved in several marker-based breeding programs to confer disease and pest resistance and to improve economically important traits (seed composition and oil quality) (Lee et al., 2007). It has immense application in the development of haploids and doubled haploids (Ramlal et al., 2023b).

### Image-based phenotyping for crop improvement

As the global demand for soybean is increasing rapidly, there is a critical need for improving soybean through breeding approaches for higher yield, enhancement of nutritional quality, and resistance to biotic and abiotic stresses. In modern breeding programs, precision phenotyping (measurement) of morpho-physiological traits along with genotyping data are considered essential inputs. Current phenotyping strategies in plant breeding have been revolutionized by advances in high-throughput phenotyping platforms that encompass imaging sensors and perform automated image acquisition, processing, and techniques for effective big data analysis, especially for the identification of superior donor genotypes and crop improvement (Das Choudhury et al., 2019; Niazian and Niedbała, 2020; Omari et al., 2020; Yoosefzadeh-Najafabadi et al., 2021). Image-based phenotyping is considered to be the most powerful tool, enabling researchers to study various plant traits at the phenome scale at multiple spatiotemporal and spectral resolutions. Due to the affordable cost of sensors and lower technical complexity, visual (RGB) sensors are mostly preferred for predicting various phenotypic traits, such as leaf area (projected shoot area), biomass (Lati et al., 2013; Parent et al., 2015; Nyalala et al., 2019), senescence (Cai et al., 2016; Wallis et al., 2022), nitrogen use efficiency in wheat (Nguyen et al., 2019), leaf area index measurement in boreal conifers (Fernandes et al., 2004), leaf chlorophyll estimation (Barman and Choudhury, 2020), nutrient uptake (Ball et al., 2020) and grain yield in corn and wheat (Gracia-Romero et al., 2019; Bekkering et al., 2020; Khaki et al., 2020, 2021; Korohou et al., 2020). Similarly, these sensors can predict growth responses to various abiotic stresses like high temperature (Grieder et al., 2015) in wheat, salinity tolerance in rice (Hairmansis et al., 2014), drought stress (Briglia et al., 2020) in grapevine, combined drought and heat in wheat (Abdelhakim et al., 2021), and disease severity of *Ascochyta* blight in chickpea (Zhang et al., 2019).

### Targeting yield attributes for improving crop productivity

Conventionally, in-plant traits such as biomass, number of tillers or nodes, number of inflorescences or flowers per node, and number of pods per plant are recorded to estimate soybean production performance. Post-harvest seed parameters, such as the number of seeds per pod, number of grains per plant, grain weight per plant, and hundred-seed weight (HSW), are required for the breeding of improved crop varieties with higher productivity. However, most of these phenotyping traits are measured visually through manual counting or low-throughput weighing scales. Traditionally, HSW is phenotyped by counting 100 seeds and measuring the fresh weight of a hundred seeds using a weighing scale. Moreover, recording the HSW of a large set of diverse crop germplasm is laborious, costly, and time-consuming, with the risk of manual errors. Recently, image-based phenotyping methodologies [image-based phenotyping in papaya (Santa-Catarina et al., 2018), high-throughput phenotyping methods for salt toxicity in lentil (Dissanayake et al., 2020), and Greenotyper (Tausen et al., 2020)] have been widely adapted to estimate both in-plant and post-harvest seed parameters, which are found to contribute to higher genetic variability to improve the productivity of diverse crops. Prediction of yield estimates from image-based measurements of canopy color (Yuan et al., 2019), number of flowers, number of grains, number of pods per plant (Lu et al., 2022), grain weight, and spike length and width (Misra et al., 2020) have been reported. Similarly, Dell’Aquila et al. (2000) reported the measurement of the area, perimeter, and length of white cabbage seeds to reveal changes in seed physical parameters during imbibition, suggesting the high potential of image analysis in seed biology studies. Digital imaging of seeds has been used to group and classify genotypes based on similarity in several crops, such as carrot (Anouar et al., 2001), ragweed (Sako et al., 2001), lentil (Shahin and Symons, 2003), and flax (Dana and Iva, 2008). Thangavel (2003) with sorghum, Kumar (2003) with lucerne, Shete (2004) with castor, Suma (2005) with sesame, and Venora et al. (2006) with *Phaseolus* sp. have used image analysis for varietal characterization. Maize tassels have been phenotyped using a tassel image-based phenotyping system for early yield prediction (Gage et al., 2017). However, no one has reported the development of an image-based method to predict the hundred-seed weight of crop plants to accelerate breeding efforts.

### Seed weight prediction using image-based seed architectural traits

Imaging and machine learning (ML) models are gaining popularity and importance for the prediction of genotypes to phenotypes that include yield, days to heading, and thousand-seed weight (Crossa et al., 2019; Khaki and Wang, 2019; Grinberg et al., 2020). Models such as random forests (RF), support vector machines (SVM), and artificial neural networks (ANN) can capture the complex interactions between genotype, phenotype, and environment in contrast to earlier methods due to their non-linearity. ML plays therefore a significant role in supporting plant breeding. ML has many applications in classical plant breeding, such as genetic diversity evaluation, yield component analysis, stability analysis, and tolerance to biotic and abiotic stresses, among others (Niazian and Niedbała, 2020), and can also be associated with analysis and prediction using omics data (van Dijk et al., 2021). Associating or predicting plant genotypes with phenotypes requires testing a series of ML algorithms, as each algorithm has its own basic assumptions and biases, and no algorithm provides the best performance for all traits. However, ML models outperform deep learning models in providing interpretive feature selection for soybean trait prediction (Gill et al., 2022).

Seed plays an important role in the life cycle of plants. Breeding new high-yielding soybean cultivars requires genetic variation in seed traits among different soybean cultivars/varieties to select superior genotypes (yield, etc.). However, information on seed morphological traits is poorly understood due to difficulties in data collection and visualization. Moreover, a major research gap is found in the identification of novel seed architectural traits (SAT) associated with the seed mass/weight trait and its genetic variation present in the large population of different soybean genotypes. Nothing has been found on the breeding value of these i-traits for their use in crop improvement. We need to decipher the dynamic relationship of these image-based SAT (i-traits) with seed weight through variance and co-variance analysis. To the best of our knowledge, this is the first study to report on image-based SAT data to identify superior donors for improving seed weight traits in soybean. The present study focuses on the development of an image-based methodology to predict the hundred-seed weight of soybean genotypes and discover superior donor genotypes with contrasting seed sizes and/or weight traits. This image-based methodology can potentially be applied to other major field crops to breed better varieties with improved traits to increase productivity.

## Materials and methods

### Plant materials

Seed architectural traits were measured from 164 different soybean accessions (including germplasm, cultivars, indigenous landraces, exotic lines, etc.; **Supplementary Table 1**) multiplied at the Soybean Genetics Unit of the Indian Agricultural Research Institute (IARI), New Delhi, India, in 2019. The freshly harvested seeds were stored in desiccators, and seed moisture content was estimated using oven-drying and gravimetric methods at different time points. Genotypes were chosen based on seed moisture content (~14%) and genotypic variation in pedigree, flower color, days to flowering, maturity duration, biomass, seed coat color, and seed yield per row. A total of 100 uniform seeds were selected from the harvested seed lot, and imaging scanners were used for phenotyping and deciphering the relationship between SAT and seed weight.

### Data acquisition

#### Image scanning setup and simultaneous accession weighing

##### Image scanning setup

**Figure 1:**
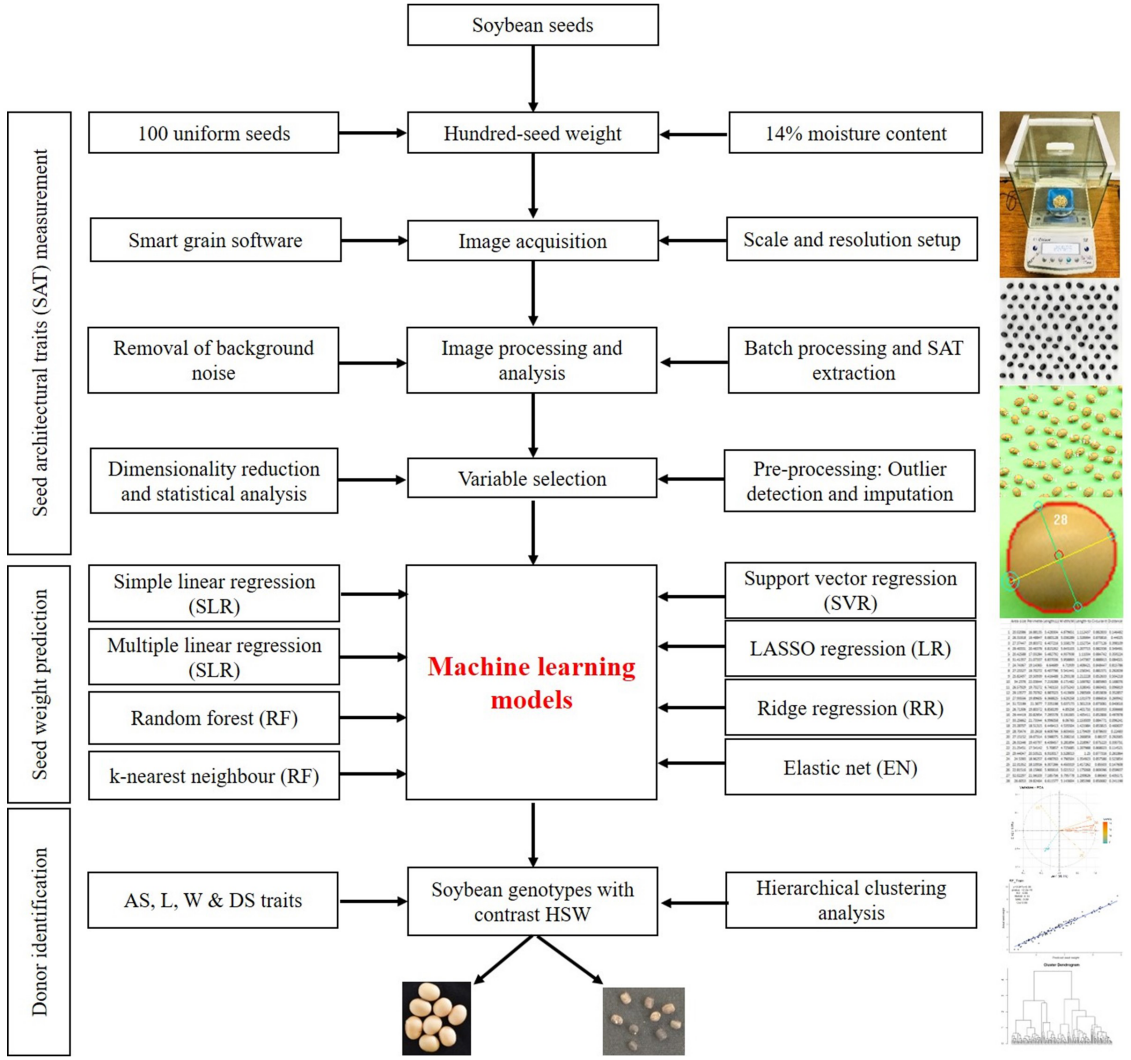
Workflow of soybean seed weight prediction using image-based architectural traits.

SATs were derived from images acquired by scanning three replications of 100 seeds from each soybean accession. Protocols described by Tanabata et al. (2012) were used for sample preparation and scanning. Seeds were arranged in a 10×10 row and column matrix on the flatbed scanning surface of the HP LaserJet scanner (model: HP LaserJetPro MFP M126nw; software version 15.0.15311.1315, released in 2021), as depicted in **Figure 1**. RGB images were captured in.jpeg format at 600 dpi with a scan area of 1024 × 1024 pixels. A 15 cm ruler was placed just to the side of the seeds during image acquisition and used as a ruler for calculating the pixel size and area (0.081 mm per pixel). Image acquisition took only a few minutes, allowing us to manually process approximately 492 batches of seeds in 2 weeks. The complete workflow of the current study related to the image-based prediction of seed weight is shown in **Figure 1**.

##### Seed weighing

Soon after image acquisition, the hundred-seed weight (HSW) was measured using a precision weighing balance (make: Mettler Toledo, model XPR204S) with up to three decimal places.

### Image processing and trait extraction

#### Image processing

SmartGrain (version 1.2), an open-source image analysis software, was downloaded (http://www.kazusa.or.jp/phenotyping/smartgrain/index.html) for image processing and analysis (Tanabata et al., 2012; Gürsoy, 2019; Leiva et al., 2022). The raw images were loaded into the software, and the developer’s recommended procedure was followed for image segmentation, processing, and data mining.

#### Trait extraction

The SmartGrain software constructs ellipses on detected grains to identify the total number of grains/seeds and also computes eight different seed architectural traits related to seed size and shape, which include umber of seeds (NS), area size (AS), perimeter length (PL), length (L), width (W), circularity (CS), length-width ratio (LWR), intersection of length and width (IS), center of gravity (CG), and distance between IS and CG (DS) (**Figure 1**). The description of the traits is length (a distance between two points around the longitudinal axis of an object and expressed in mm), width (measured in the horizontal X-axis/transverse axis and expressed in mm), perimeter length (multiplication of the length, width, and height of the object and expressed in mm), area (multiplication of the length and width of the object and expressed in mm^2^), and circularity (square root of the ratio of the actual area of the object to the area of a circle with the same circumscribed circle: where A is the actual area of the object, AP is the area of a circle with a diameter equal to the circumscribed diameter (or) length of the object). DS is the distance between IS and CG and represents the shape of the seed.

### Data analysis

#### Data preprocessing

##### Outlier detection and imputation

Outliers and missing values in the data can influence prediction accuracy. Hence, data obtained from an image-based assessment of selected accessions were first subjected to missing value and outlier detection and imputation using the RStudio program (V: 2022.02.3). A multivariate approach was used to detect outliers, where box plots were generated for the continuous variable and observations outside 1.5 times the interquartile range (the difference between the 75^th^ and 25^th^ quartiles) were considered outliers and imputed with the mean of other replications. 

##### Data normalization (z-score scaling)

The dataset contained variables with different units from pixel to weight in mg, area in cm^2^ and length in mm, therefore, to use the complete data frame for statistical analysis, it was imperative to scale it to a single range. We selected a simple feature scaling method, standard z-score (*mu*), which used variable averaging and standard deviation as factors to normalize all variables close to zero and in the range from -3 to +3. The z-score feature scaling method was selected as it takes into account both the mean value and the variability in a set of raw scores.

### Statistical analysis and visualization of results

The pre-processed clean data obtained through image-based assessment of selected accessions were subjected to various statistical analyses, such as descriptive statistics analysis, analysis of variance (one-way), calculation of Tukey’s HSD, broad sense heritability and components of variance (genotypic, phenotypic, and environmental coefficient of variance), coefficient of variance and its components of variance (genotypic, phenotypic, and environmental coefficient of variance) using the variability package (Briglia et al., 2020) in Rstudio. *Psych library* (Mohi-Ud-Din et al., 2021) was loaded in Rstudio, and the pairwise panel command was used for visualization of Pearson’s correlation coefficient (r) and plotting frequency distribution. A hierarchical clustering of genotypes was performed to group the similar genotypes into clusters using the hclust command with two complementary methods: the average linkage hierarchical clustering method (ALHCA) and the complete linkage hierarchical clustering method (CLHCA). The members of clusters were compared between the two methods, and a confusion matrix was formed to revalidate the clustering of member genotypes based on image-based seed traits.

### Feature selection, identification of superior traits, and donor genotypes

#### Trait suitability analysis using the variance inflation factor

The multiple independent variables and their relationships with each other greatly influence the prediction accuracy of dependable variables. Hence, trait suitability analysis was performed by estimating the variance inflation factor (VIF) using the library car and the VIF command in Rstudio. The VIF is a measure of the amount of multicollinearity in a set of multiple regression variables. A VIF value greater than 10 was considered to have very high multicollinearity and was removed or reconsidered from further analysis.

#### Superior image feature (i-trait) selection

Two methods of feature selection were employed. First, principal component analysis (PCA) was used, which is an unsupervised method of feature selection that does not use information about the feature value and target value. The PCA also used normalized data, which requires some kind of transformation before analysis. Hence, we used an additional method called the Boruta algorithm, which used wrapper methodology for the accurate identification of superior image features for seed weight prediction. The PCA was performed in RStudio using the *FactoMineR library* (Mohi-Ud-Din et al., 2021), and the scree plot was constructed using the fviz_screeplot function. The first two principal components were selected based on the elbow angle of the scree plot. The PCA biplot was constructed with facto extra using the fviz_pca_biplot function. The 10 best image features contributing the most variance to the PC 1 and PC 2 components of PCA were plotted as a bar chart using the fviz_contrib function. Similarly, the 10 best soybean genotypes were selected based on the highest variance contribution to the PC 1 component and plotted as a bar chart. The Boruta function was used in RStudio with raw data set to max run 500. With this method, a subset of features called shadow features was used to train a model. The model developed from the shadow features was used to select the feature based on forward or backward selection elimination. Finally, the features were listed based on their importance, with the highest MaxImp value and decision.

#### Trait prediction (seed weight as a dependable variable) using ML models

To predict soybean weight based on the extracted feature variables, seven regression models were explored in this study: simple linear regression (SLR), multiple linear regression (MLR), random forest (RF), support vector regression (SVR), lasso regression (LR), ridge regression (RR), and elastic net (EN). The caret (Yuan et al., 2019), mlbench (Elangovan et al., 2023), and glmnet (Singh et al., 2023) packages were used to train the machine learning models in RStudio. To predict soybean seed weight, a dataset comprising seven image-based architectural traits derived from 164 different soybean genotypes was partitioned into (80:20%) a training (131) and a test (33) dataset for validation. All seven regression models were repeated 10 times with cross-validation on the training data using the trcontrol function with the repeated cv method. A linear regression model was constructed using the training dataset, using SW as a dependent variable and all other image features together or separately as independent variables. The lm, rf, and svmLinear2 functions were used to build linear, random forest, and support vector machine models, respectively. The eps regression type was used with the linear kernel type and cost=0.1 for SVM models, and the tuning values were 0.001, 0.01, 0.1, 1, 10, and 100. A ridge regression model was performed using the glmnet package with tunegrid alpha=0, lambda=0.0001, and trcontrol with 10 times validation. A lasso regression was performed using the glmnet package with tunegrid alpha=1, lambda=0.0001, and trcontrol with 10 times validation. An elastic net regression was performed using the glmnet package with tunegrid alpha=0.1, length=10, lambda=0.0001, and trcontrol with 10 times validation. Knn models were performed using the caret, pROC, and mlbench packages with a k value=1:70.

### Comparison of ML model performance and the best model for predicting SW

The predict function was used to forecast the SW value of the testing dataset based on a model developed on the training dataset. The coefficient of determination (R^2^), root mean square error (RMSE), and mean absolute error (MAE) were used as evaluation metrics to quantify the performance of the regression models and to determine how well the model predicts new data and whether the model is too complicated. The cor function was used to estimate Pearson’s correlation coefficient between the predicted SW value and the actual SW value in the testing dataset. The lm function was used to measure the coefficient of determination (R^2^) between the predicted and actual SW values in the testing data set. The root mean square error (RMSE) was calculated from the square root of the mean estimated from the difference between actual and predicted SW in the training dataset. The mean absolute error (MAE) was calculated from the mean absolute difference between the predicted and actual SW of the training dataset. The ggplot2 package was used to plot the regression model graphs of the predicted and actual datasets.

## Results and discussion

The study generated ~ 24.6 GB of high-resolution RGB image data by scanning the seeds of 164 different soybean genotypes. The processed image data included the data matrix array of 1,14,737 data points related to eight SATs (NS, AS, PL, L, W, LWR, CS, and DS) estimated from 100 individual seeds of each of the 164 soybean genotypes. We removed NS from further analysis because it was 100 and common to all of the 164 different genotypes. The data preprocessing efforts identified approximately 763 data points that were either missing or found to be outliers, hence statistically imputed to have a complete data matrix array of 1,15,500 data points for further analysis. Finally, the SAT data collected from 100 seeds of each genotype were pooled, and the mean values were analyzed along with the hundred-seed weight. A very low coefficient of variation (<5%) was found for all SAT collected from 100 individual seeds of each genotype (**Supplementary Table 2**). This suggested that the 100 seeds selected for imaging have uniform and pooled average values represent the SAT characteristics of each genotype while the descriptive statistics and ANOVA results are presented in **Table 1**.

**Table 1:** The mean and associated standard error ( ± SE), maximum, minimum, least significant differences (LSD_0.05_), estimated genotypic (S^2^g), and experimental error (S^2^e) variance components, among the soybean germplasm accessions.

SoV	SA	PL	L	W	LWR	CS	DS	SW
Minimum	13.36	13.89	4.48	3.69	1.07	0.77	0.20	4.85
Maximum	24.04	19.18	6.35	5.75	1.33	0.91	0.56	9.46
Mean ± SE	18.09 ± 0.19	16.32 ± 0.13	5.29 ± 0.06	4.47 ± 0.005	1.19 ± 0.005	0.85 ± 0.001	0.29 ± 0.001	6.69 ± 0.06
C.V.	12.47	6.72	6.33	7.66	3.15	2.61	14.94	13.70
C.D 5%	0.52	0.37	0.15	0.01	0.02	0.03	0.003	0.18
F value	141.61***	64.60***	35.07***	4540.4***	46.67***	1.91***	1403.7***	208.79***
Heritability	0.98	0.96	0.92	0.99	0.93	0.20	1.00	0.97
GV	4.98	1.15	0.10	0.11	0.0013	0.0001	0.002	0.83
EV	0.11	0.05	0.01	0.0001	0.0001	0.0004	0.00001	0.01

*** Significant at p<0.001, surface area (SA), perimeter length (PL), length (L), width (W), length-to-width ratio (LWR), seed circularity (CS), and distance between IS and CG (DS) and hundred-seed weight (SW).

**Figure 2:**
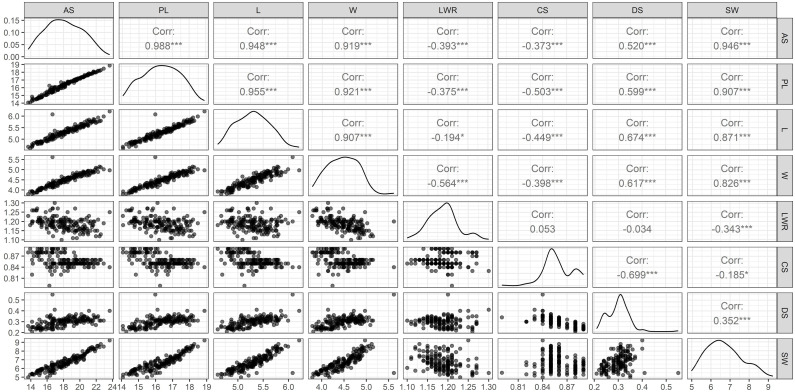
Pair’s panel matrix depicting frequency distribution, regression, and correlation coefficient (r) of eight different SAT measured from 164 soybean genotypes. Correlation coefficient at the significant level of at P<0.05 (*) and P<0.001 (***). Surface area (SA), perimeter length (PL), length (L), width (W), length-to-width ratio (LWR), seed circularity (CS), distance between IS and CG (DS), and hundred-seed weight (SW).

The ANOVA results showed statistically significant genotypic variation in selected different soybean genotypes for seed architectural traits and hundred-seed weight (*P* ≤ 0.001). The presence of genetic variability for seed traits such as seed length, width, and weight has been reported using conventional phenotyping methods. Imaging technology has advantages over conventional phenotyping as it records more trait resolution (SA, PL, L, W, LWR, CS, and DS) per unit of data recording time. Kim et al. (2022) demonstrated the use of eight different seed architectural traits to predict seed weight in soybean. In that study, apart from architectural traits, seed coat color was also documented for diversity characterization of germplasm according to UPOV classification. Recently, several pieces of literature reported the variability of seed traits captured using imaging sensors in soybean (Yuan et al., 2019; Baek et al., 2020; Yang et al., 2021; Lu et al., 2022). However, the present work demonstrated the application of imaging sensors to predict SW from SAT, which is an important yield-contributing trait to identify superior donors for breeding better soybean crop plants. Pair's panel matrix analysis clearly showed that all eight SATs data following the normal distribution pattern (**Figure 2**). Hence, these traits are expected to show polygenic inheritance and be controlled by multiple quantitative trait loci. Genetic variability plays a significant role in plant breeding, and the extent of heterogeneity in germplasm is governed by genetic variation (GV) and phenotypic variation (PV) of traits. For all attributes, the variance component for error (environmental variation) was lower, indicating that selection is useful in breeding these traits (**Table 1**). Heritability represents the phenotypic variation present in a population that is mainly contributed by genetic variation between individuals in that population. Among the SATs, size-related traits (AS, PL, L, W, and DS) showed very high (>95%) broad sense heritability, along with SW (0.97). The lowest heritability value (0.20) was observed for the shape-related SAT (CS).

### Seed size features determine seed weight

**Figure 3:**
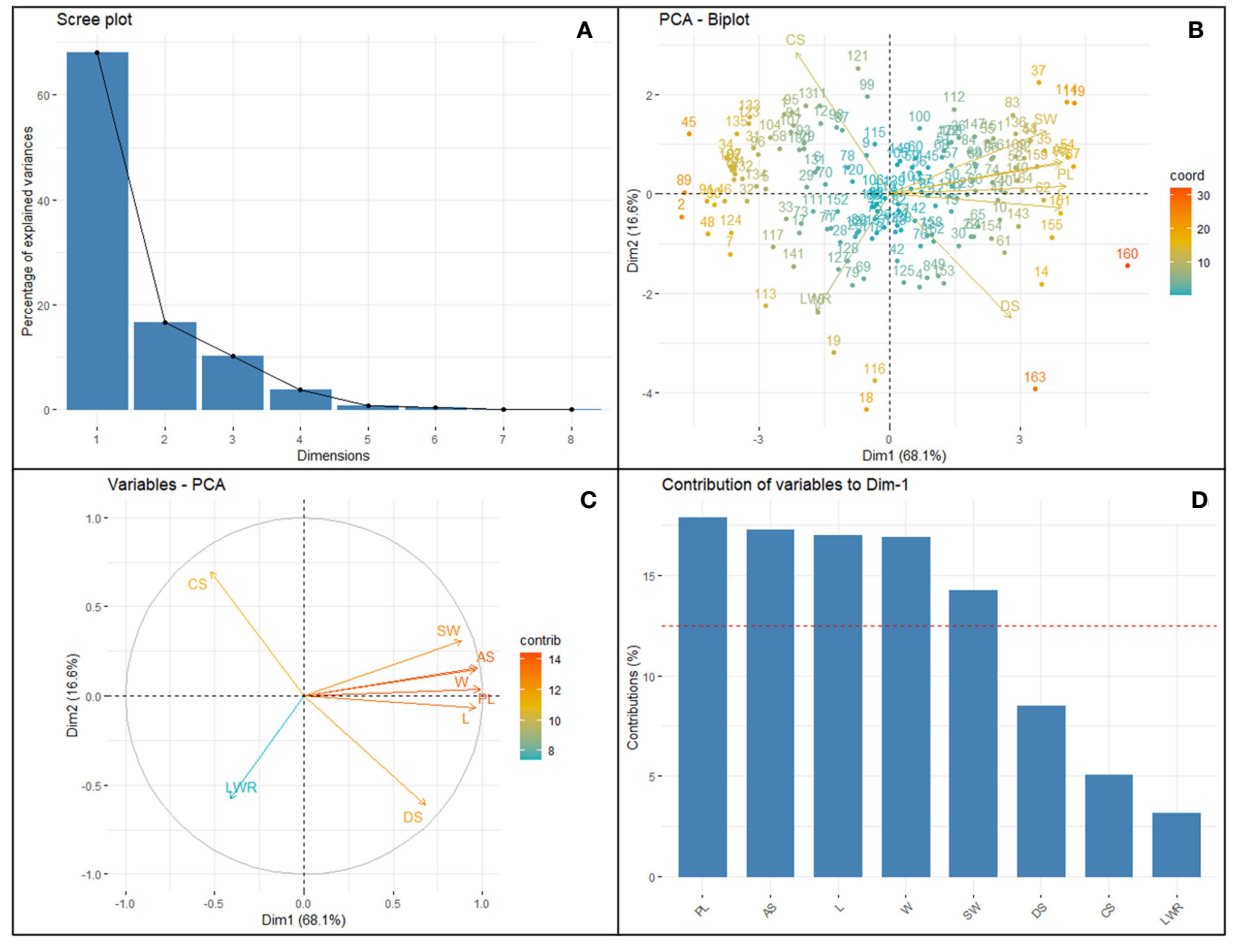
Principal component analysis of eight selected SATs with CVs ≥0.3 collected from 164 different soybean genotypes. Clockwise from the top left panel **(A)** Scree plot depicting the number of informative principal components (2). Top right **(B)** bi-plot showing the distribution loading vectors and position of each genotype in the PC space; Bottom left **(C)** panel shows the relationship and importance of the traits among each other; Bottom right **(D)** the importance of the traits in terms of the highest contribution of variance to the principal component PC1 vs. PC2, representing 84.74% of the variability. Surface area (SA), perimeter length (PL), length (L), width (W), length-to-width ratio (LWR), seed circularity (CS), and distance between IS and CG (DS) and hundred-seed weight (HSW).

The selected i-traits related to seed size (AS, PL, L, W, and DS) showed a strong and significant positive Pearson correlation coefficient (P<0.001) with SW (**Figure 2**). These traits can potentially be used for breeding better soybean plants by predicting SW and selecting superior donors for crop improvement. Although seed shape-related traits (LWR represents the ovality of seed shape) showed a very high heritability (0.93) in our population, Pearson’s correlation coefficient showed a significant negative correlation between SW and LWR. Another shape-related trait (CS) showed the lowest broad-sense heritability (0.20) and Pearson’s correlation coefficient (-0.19) with SW. In the case of DS, the highest heritability (1.00) and moderate positive correlation (r=0.35) between SW and DS were recorded. These results clearly showed that the seed shape and size of different soybean genotypes were extremely variable, ranging from round to elliptical. The seeds of most of the cultivars used in this study were oval, with the highest genetic variation towards ovality. However, shape-related traits (neither ovality nor circularity) hardly influence SW. Among the SAT, the highest correlation value (r=0.99) was found between SA and PL. Kim et al. (2022) have reported similar higher positive correlation between the SA and PL. SW was positively correlated with SA, PL, L, W, and DS and negatively correlated with LWR and CS. The PCA bi-plot also showed that seed shape (DS and LWR) and size-related traits (AS, L, W, PL, and DS) were negatively correlated, and the loading vectors were grouped in the opposite diagonal quadrants (**Figure 3**). The seed size-related traits were identified as a major positive contributor to the variance in PC1 and PC2 components. In contrast, seed shape-related traits were found to be the negative contributors to the PC1 and PC2 components of the biplot. Thus, seed size determines the seed weight more than seed shape traits (Cober et al., 1997; Kim et al., 2022) in soybean.

### Image feature selection and superior trait identification

Trait suitability analysis was performed using variance inflation factor (VIF) analysis. It was used to measure the degree of multicollinearity among the independent variables in multiple regression models. In general, multicollinearity does not reduce the predictive power of multiple regression models, but it does reduce the statistical significance of independent variables. A large VIF (>10) of an independent variable indicates a highly collinear relationship among the independent variables (**Supplementary Table 3**). Among the traits, the VIF value ranged from 1.47 to 18.90. Very high multicollinearity was observed to exist between AS and PL, 18.90 and 17.21, respectively. Although both AS and PL recorded >10 VIF, AS was selected as suitable for multiple regression model analysis due to its technical advantage over PL. This is because predicting the seed weight based on the mass per pixel has advantages over simple boundary pixel count (PL) estimation, as the latter has a higher probability of overestimating or underestimating and detecting outliers.

Similarly, feature selection is an important step in a data set with many variables and features. Boruta analysis eliminates unimportant variables to improve the accuracy and performance of the model. Boruta adds randomness to the given data set by creating shuffled copies of all features, called shadow features, and determining at every iteration whether a real feature has higher importance than the shadow feature. It simply reduces dimensions and training time. Boruta allows feature selection and ranking based on the Random Forest (RF) algorithm. Seven features recorded by imaging were subjected to Boruta analysis in all features that recorded values for mean, median, and maximum importance and also remained beyond shadowMax (**Supplementary Table 4**). AS was the best predictor variable with the highest maximum importance (27.94), followed by L (22.15) and DS (20.86), while LWR (4.71) and CS (9.90) were the least important in predicting SW. The Boruta plot clearly showed that all seven SAT features had a higher Z-score than the maximum feature of the shadow feature, and constancy removed unimportant features (**Supplementary Figure 1**).

### PCA biplot

The purpose of the PCA is to obtain a small number of linear combinations of the eight variables that account for most of the variability in the data. The Principal Component Analysis describes the total variation available in the population and the relative distance among the 164 genotypes for each combination of seed traits studied. So, the most informative trait can be selected through dimensionality reduction and data normalization techniques. In this case, two components were extracted, and the inclusion criterion used was that two components had eigenvalues greater than or equal to 1.0 (**Supplementary Table 5**). This result indicates that the variability of the seed traits studied by imaging soybean seeds is mostly explained by PC1 and PC2 of symmetrical variation. PC1 vs. PC2 accounted for 84.74% of the variability in the loading plots. PCA of the accession-by-trait correlation matrices was performed, and a biplot was generated to allow the clustering of genotypes (**Figure 3**). The biplot showed both the loadings of each variable (arrows) and the scores of each genotype (numbers). The angles between the arrows show their close association in terms of correlations. The traits AS, L, PL, W, and SW showed a positive association (angles between the directional vectors <90°). The first component (PC1) accounted for 68.11% of the variability and primarily included image-based seed size traits such as PL, SA L, W, DS, and manually recorded SW traits. PC2 accounted for 16.62% of the variation derived from CS, SA, PL, W, and SW. A wider angle of loading variable was found for LWR and SW for both PC1 and PC2 components on a negative slope. This indicated that LWR had a significant negative relationship with soybean hundred-seed weight. The genotypes located closer in the plot showed similar scores on the PCA components and exhibited similar profiles of image traits. All genotypes were distributed in all quadrants and showed no distinct separation. The loading score of the PC1 vs. PC2 plot with the largest PL, SA, L, W, DS, CS, and SW (**Supplementary Figure 5**) was identified as the number of superior genotypes (**Supplementary Table 5**). Eight seed traits with CVs ≥0.3 (**Table 1**) were used for factor analysis using the PCA extraction method. For each trait, the largest variable loading score in PC1 and PC2 is shown in bold. Among the traits, PL, AS, L, W, DS, and SW were identified as the highest variance (>12.5%) contributing variables and were found to be important features in the SAT. CS and LWR are the lowest variance-contributing variables recorded in the population. Kim et al. (2022) reported a similar trend of results.

### Comparison and identification of the best prediction model to predict SW

Simple linear regression models were performed with each of the selected independent variables (AS, PL, L, W, LWR, CS, and DS) separately on the dependent variable (SW), and the results are presented in **Table 2** and **Supplementary Figure 2**. The models were trained using 80% of the data (131), and the linear prediction model was tested on 20% of the data (33). The prediction accuracy (R^2^), Pearson’s correlation coefficient (r), statistical significance (p-value), root mean square error (RMSE), and mean absolute error (MAE) were estimated between the predicted SW (pSW) and the actual seed weight (aSW; ground truth). In both the training dataset (TrD) and the testing dataset (TsD), the results showed a high positive correlation (>0.80) between aSW and pSW using AS, PL, L, and W with a high statistical significance (p>0.001) (**Supplementary Figure 3**). This showed that seed size-related traits were more informative for predicting seed weight than seed shape factors. Among the dependent variables, AS was found to be the best predictor with the highest coefficient of determination/prediction accuracy (R^2 =^ 0.89) in both TrD and TsD (**Supplementary Figure 7**). The model equations of LWR, CS, and DS showed weak correlation (<0.50) between aSW and pSW, high RMSE and MAE values (>0.70), and prediction accuracy (R^2^<0.1). Therefore, it was not found to be suitable for the prediction of SW. Next to AS, PL was identified as the next best predictor, with an accuracy of R^2 =^ 0.82 in TrD and R^2 =^ 0.80 in TsD. Among these two predictor traits, AS was the best choice owing to the lowest RMSE (TrD= 0.39; TsD=0.27) and MAE (TrD=0.32; TsD=0.22) values in both TrD and TsD. Moreover, SW prediction using AS was observed to be more accurate as the RMSE (0.27) and MAE (0.22) values of TsD were significantly lower than TrD (RMSE=0.39; MAE=0.32). Singh et al. (2020) reported that a simple foreground area was found to be the best predictor and has a greater association with seed weight. A similar type of image-based seed weight prediction using seed architectural traits has been reported in rice (Singh et al., 2020), wheat (Sabanci et al., 2016), and forest species (Felix et al., 2021).

**Table 2:** Performance indicators of simple linear regression models used to predict SW using selected SATs as independent variables (AS, PL, L, W, LWR, CS, and DS).

SNo	Formula	Training set						Testing set					
		Equation	R	P	R2	RMSE	MAE	Equation	R	P	R^2^	RMSE	MAE
1	SW ~ AS	SW=0.89XAS+0.71	0.90	<0.001	0.89	0.39	0.32	SW=0.89XAS+0.77	0.95	<0.001	0.89	0.27	0.22
2	SW ~ PL	SW=0.82XPL+1.18	0.90	<0.001	0.82	0.39	0.32	SW=0.77XPL+1.52	0.90	<0.001	0.80	0.36	0.28
3	SW ~ L	SW=0.75XL+1.62	0.87	<0.001	0.75	0.46	0.32	SW=0.68XL+2.12	0.88	<0.001	0.76	0.41	0.31
4	SW ~ W	SW=0.65XW+2.31	0.81	<0.001	0.65	0.54	0.37	SW=0.77XW+1.55	0.90	<0.001	0.81	0.36	0.30
5	SW ~ LWR	SW=0.11XLWR+5.95	0.33	<0.001	0.10	0.86	0.69	SW=0.06XLWR+6.30	0.21	0.004	0.04	0.94	0.75
6	SW ~ CS	SW=0.01XCS+6.56	0.12	0.013	0.012	0.90	0.73	SW=0.006XCS+6.63	0.06	0.58	-0.007	0.97	0.80
7	SW ~ DS	SW=0.09XDS+6.01	0.31	<0.001	0.095	0.86	0.67	SW=0.11XDS+5.99	0.44	<0.001	0.185	0.89	0.72

Pearson’s correlation coefficient (r), Root mean square error (RMSE); Coefficient of determination (R^2^); Mean absolute error (MAE).

**Figure 4:**
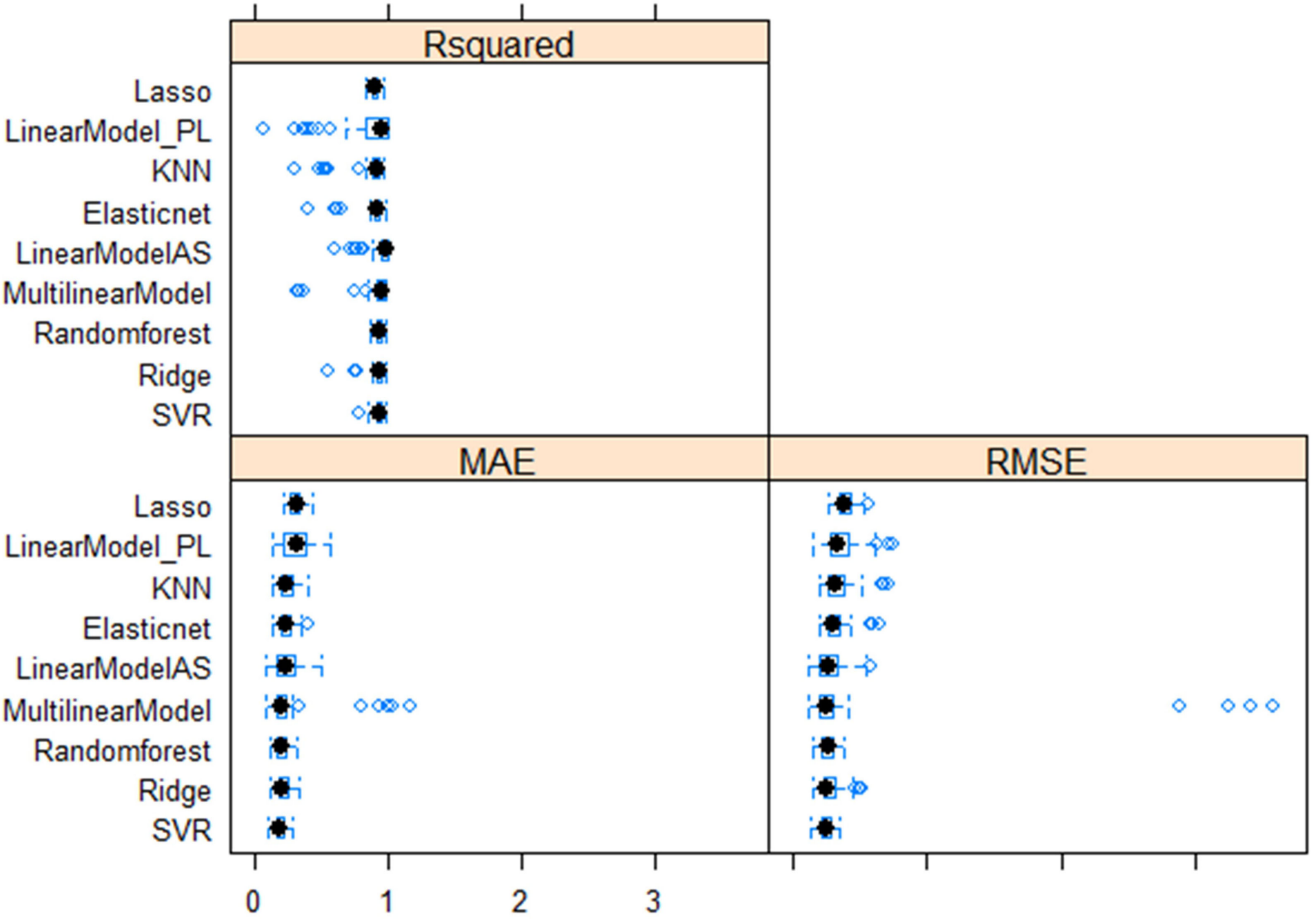
Comparative performance of nine machine learning models (LASSO regression, Least Absolute Shrinkage and Selection Operator; Linear_PL, Linear regression model using PL as the independent variable; KNN, *k-nearest neighbors* algorithm (k-NN); Elastic net regularization; Linear_AS, Linear regression model using AS the independent variable; MultilinearModel, Multiple linear regression model using seed size factors; i.e., AS, L, W, and DS as the independent variable, Random forest regression, Ridge regression, Support vector regression models.

**Figure 5:**
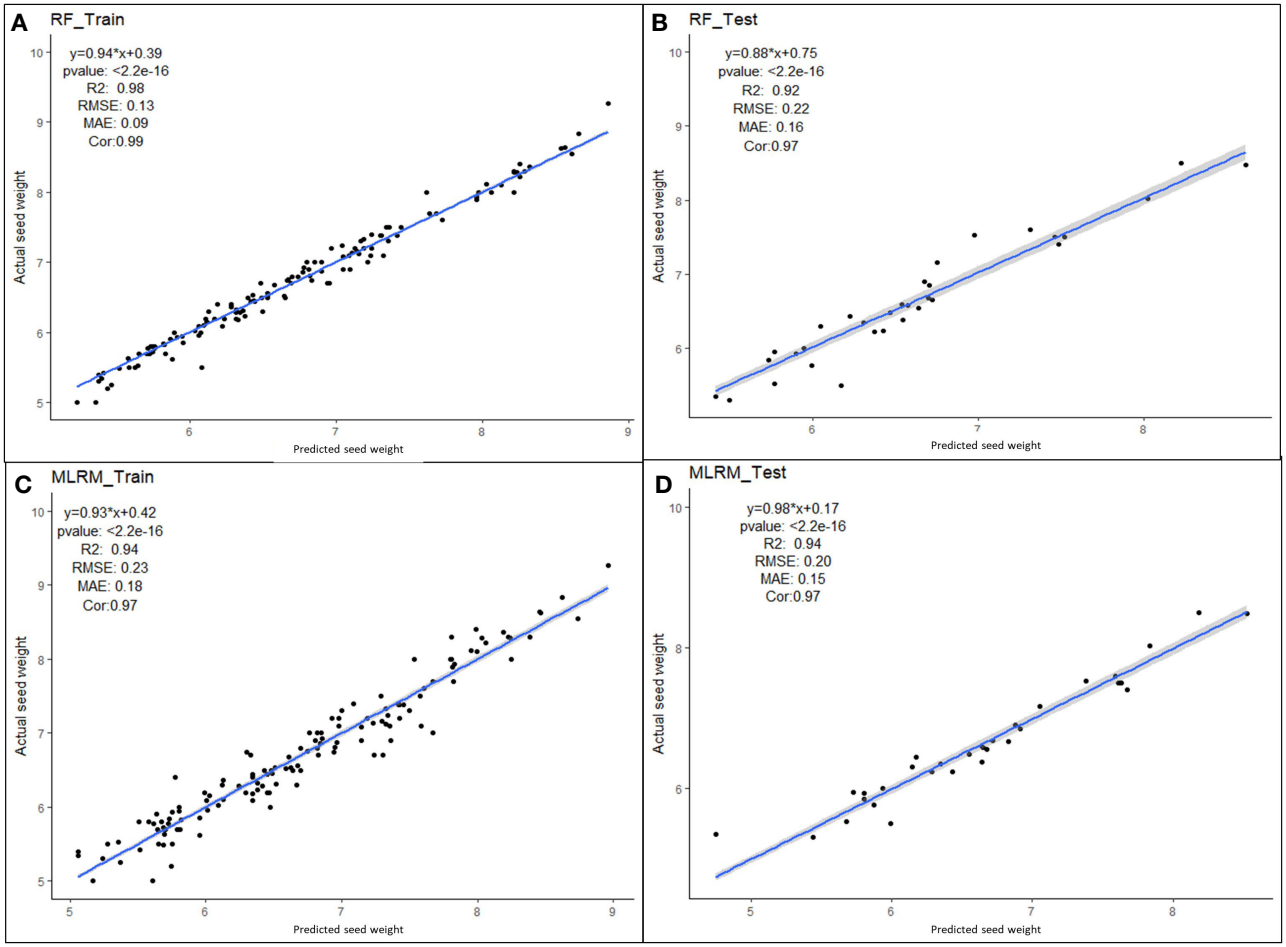
Performance of superior machine learning models for predicting seed weight using seed size factors (AS, L, W and DS). Prediction models using **(A)** RF training dataset, **(B)** RF testing dataset, **(C)** MLR training dataset and **(D)** MLR testing dataset.

Multiple linear regression models were performed with four superior independent variables (AS, L, W, and DS) on the dependent variable (SW), and the results are presented in **Supplementary Table 7**. Irrespective of the multivariate regression models (MLRM, RF, SVR, Lasso, Ridge, EN, and KNN), the results showed a high positive correlation (>0.90) between aSW and pSW in both the training dataset (TrD) and the testing dataset (TsD) (**Figure 3**). Among the models, RF was found to be the best model with the highest coefficient of determination/prediction accuracy (R^2 =^ 0.98) in TrD and the lowest RMSE (0.13) and MAE (0.09). Next to RF, the MLRM model was found to be the best model with a high R^2^ (0.94), low RMSE (0.23), and MAE (0.18). MLRM was found to be the best predictive model for TsD with R^2^ (0.94), RMSE (0.20), and MAE (0.15). In TsD, RF was the next best predictor with an accuracy of R^2^ (0.92), RMSE (0.22), and MAE (0.16). The model equations of Lasso, KNN, and EN recorded relatively low predictive accuracy (R^2^ < 0.1), high RMSE, and MAE values (>0.15), and were therefore found to be unsuitable for predicting SW. Among the predictor models, RF and MLRM stood out as the best choices owing to the highest prediction accuracy, and correlation with the lowest RMSE and MAE values in both TrD and TsD (**Figures 4**, **5**).

Morphology is an important and effective attribute and a useful feature to discriminate objects that vary with mass in image-based crop phenotyping (Khoshroo et al., 2014). Several reports digitally determine the component traits of SAT, namely, area (AS), perimeter length (PL), length (L), width (W), length-width ratio (LWR), circularity (CS), center of gravity (CG), intersection of length and width (IS), and distance between IS and CG (DS), using RGB images (Tanabata et al., 2012). There are many Windows- or Android-based free open-source software products used to measure grain size and shape, such as SmartGrain (Tanabata et al., 2012), GrainScan (Whan et al., 2014), and ImageJ (Abràmoff et al., 2004) for digital phenotyping of SAT. A few paid commercial software products, namely Lemnagrid (LemnaTec scanalyzer 3D), can be utilized to increase the trait resolution of SAT phenotyping. It is very simple and easy to capture the images with scanners to extract seed properties (Tanabata et al., 2012). Although several reports have been published for the non-invasive prediction of whole plant biomass using pixel counts derived from digital images, very little information is available for predicting hundred- or thousand-seed weights of soybean genotypes through SAT.

Recently, Sabanci et al. (2016) used a Logitech c905 webcam camera to acquire still images and accurately predicted wheat seed weight (99.66%) using a simple polynomial regression model. Similarly, Felix et al. (2021) determined the thousand seed weight (TSW) of seeds harvested from 16 different forest tree species using digital image processing through image J software and demonstrated the comparative advantages (60-80% time savings) of image-based phenotyping for counting seed number, weight, and TSW over traditional manual measurement. Singh et al. (2020) used a mobile camera (Apple iPhone) to acquire the RGB images and used six machine learning algorithms in a stacked ensemble model to predict the size and weight of rice kernels using the pixel count derived from the segmented foreground images. In all of the above cases, the prediction accuracy was very high (R^2^ > 0.95), either because of the smaller sample size or because of the application of powerful machine learning models to predict seed size and weight.

Recently, Lu et al. (2022) used 24 soybean varieties and used deep learning neural network techniques such as generalized regression neural network (GRNN) and You Only Look Once (YOLOv3) algorithms to predict grain yield per plant (with 90.3% prediction accuracy) using leaf number count as an independent variable and pod categorization based on the number of seeds per pod in hidden layers. Similarly, Rajković et al. (2021) used ANN and RFR models to predict six major crop production-oriented traits such as oil content, seed yield, protein content, oil and protein content, thousand seed weight, yield, and quality of rapeseed. They used 40 different soybean genotypes and found RFR models to be the best model (0.88% prediction accuracy) to predict the best year of production and genotype. Yuan et al. (2019) used early-season canopy RGB images to predict soybean yield, maturity, and seed size using 457 dependable variables related to color and texture features. Prediction accuracy was limited to 0.58% for seed size and 0.62% for yield using cubist and random forest regression models. Singh et al. (2020) used stacked ensemble models comprising ANN, RF, SVM, KRR, and KNN for grain size and weight prediction. They found that the normalized pixel area of the rice kernel predicts the single kernel weight with 0.95% accuracy. Smitchger and Weeden (2018) found the ideal seed size for maximum seed yield in pea plants and reported that the seeds with the maximum size had the highest seed weight and harvest index. However, in all these cases, the main focus was on in-field plant traits to predict the soybean seed weight. However, no previous study has reported using seed architectural traits to predict seed weight. This is the first report to predict the hundred-seed weight using multiple image-based seed architectural traits and machine learning algorithms.

Hundred-seed weight is a very important trait that physiologists and breeders measure to record and maintain the pedigree and yield performance of the genotype. Conventionally, automatic seed counters that work on the principle of vibrator and phototransistor sensors are widely used in the seed industry and by scientific organizations for seed counting. Traditionally, 100 seeds were counted either manually or by machine and then weighed manually using a scale to measure HSW. Recently, a company called Elmor combined a grain counting and weighing machine for the determination of the thousand kernel weight. Through the use of this modern machine, there has been a significant reduction in manual labor efforts. However, the time required for counting and weighing remains a major bottleneck in traditional phenotyping methodologies. It is advantageous to have image-based HSW estimation tools for the automatic measurement of HSW and simultaneous measurement of seed weight using seed architectural traits, such as color traits, of each seed. There is a crucial need to enhance the productivity of soybean plants by breeding for uniform, larger seed sizes per plant. This will result in higher yields and facilitate mechanical harvesting. Moreover, the image-based technology has the added advantage of mapping quantitative trait loci (QTL)’s/genes from seed architectural traits estimated using multiple numbers of seeds harvested from a large number of genotypes. Researchers spend much of their productive time counting and weighing the seeds from a large number of different genotypes. Hence, there is a need for faster, automated image-based phenotyping tools for estimating HSW. Generally, seed weight prediction using imaging techniques uses white light-illuminated platforms, and the major bottleneck associated with causing an error is related to the underestimation of closely located overlapping seeds. Placing seeds in a non-overlapping pattern on the scanning platform took more time in our case. In such cases, tools that automatically place seeds through vacuum tube techniques, predict hundred-seed weight based on a lower number of randomly placed seeds, or use accurate image processing techniques that take care of the overlapping seed count or the sequential imaging of seeds using push broom conveyor systems are the basic engineering needs to be met for the invention of automatic HSW machines. Sabanci et al. (2016) used a variable number of 25 to 100 grains of wheat and predicted the seed weight at a 99.66% R^2^ value, even with a smaller number of seeds. The mass of the seed is determined by its dry matter and moisture content. With the recent advent of RGB and NIR sensors, multi-modal regression algorithms can be developed to predict seed moisture content using the NIR signal and the weight component using the RGB image-derived AS feature. Several smartphone-based Android mobile applications (seed counter and seed counting) are available to estimate various seed architectural traits. This holds promise for the future development of an agronomically user-friendly mobile application that accurately predicts seed weight using SAT. Thus, the enormous labor, time, cost, and space involved in conventional phenotyping can be minimized to hasten the breeding cycle. In this experiment, we demonstrated the image-based methodology to predict the breeder’s best traits of interest (hundred-seed weight) for improving the productivity of soybean genotypes using image-based architectural traits. The contrast donor genotypes were effectively used for bi-parental crossing and mapping of QTLs associated with seed size and weight traits. This methodology can be easily deployed in other major cereal and pulse crops to breed better genotypes with higher productivity.

### Donor selection based on superior i-trait and hierarchical clustering analysis

The identification of soybean donor genotypes contrasting in seed size, shape, and weight was the main objective of the current experiment. Among 164 different soybean genotypes, HSW was highest in JS 20-30 (9.24g), DS 9712 (8.81g), JS 93-06 (8.61g), PK564 (8.59g), JS9423 (8.52g) and UPSL-332 (8.54g). In contrast, HSW was lowest in BJJF-8 (4.9g), UPSL-365B (5.24g), M-53 (5.29g), L-768 (5.29g), UPSL-736 (5.33g), UPSL-326 (5.233g) and L-780 (5.38g). The superior i-traits (AS) closely associated with SW were found to be highest in soybean genotypes like JS 20-33 (23.65), JS9423 (22.91), DS9712 (22.56), UPSL-332 (22.41), JS 93-06 (22.23) and PK564 (22.17). Genotypes like BJJF-8 (13.67), L-768 (13.92), L-780 (14.15), UPSL-326 (14.15), UPSL-736 (14.24), and M-53 (14.37) were found to have the lowest SA. These genotypes were also identified to follow a similar trend in the case of size and shape-related traits (PL, L, and W) (**Supplementary Table 8**). The presentation of the results of Tukey HSD clearly showed that these contrasting soybean genotypes were statistically different and assigned different alphanumeric values with the help of the *multcomView* function in RStudio. The data on seed size and weight diversity clearly showed that the panel used in this study were mostly indigenous germplasm seeds, and, as reported earlier, the exotic lines (Chinese and Korean) soybean seeds were found to possess larger seed size and weight (Kim et al., 2022).

The divisive hierarchical clustering analysis was performed to arrange all the observations of eight variables collected from 164 genotypes. The clustering started with all observations in one cluster at the top, and then splits were performed recursively as one moved down in a top-to-bottom approach. The hierarchy was performed based on a metric called distance, and a linkage criterion was specified as the pairwise distances of observations in the sets. Two appropriate linkage criteria (ALHCA and CLHCA) were performed using the mean and maximum distance between elements of each cluster (**Supplementary Figures 4A, B**). The confusion matrix (**Table 3**) clearly showed that the first and second clusters comprised 66 and 26 genotypes, respectively, that had similar SATs within the cluster based on the distance matrix calculated in both methods of HCA. Similarly, the third and fourth clusters were found to contain 49 and six genotypes with more similar trait combinations, respectively. Out of 164 different soybean genotypes analyzed, 17 genotypes were grouped in the third cluster by CLHCA and in the fourth cluster by ALHCA in the confusion matrix (**Table 3**). Thus, the HC, or grouping accuracy, of 89.63% was achieved by using all eight different SATs for the identification of donors. Among the clusters, Cluster 1 was the largest, consisting of a large number (66) of genotypes. The list of cluster group members is presented in **Supplementary Table 9**. The contrast donors for soybean genotypes for seed size and weight were selected from clusters 2 and 4. Kim et al. (2022) reported the genetic diversity of soybean seed weight in the range of 8 to 24 g. In our case, the cluster-wise range and mean values of SW were in the range of 5 to 9 g (**Table 3**). This showed that the selected set of germplasm is not sufficient to capture the genetic variability for seed size and weight. However, the same methodology can be explored in more technically recognized core germplasm sets. The highest means for both AS and SW were observed in Cluster 4, and the lowest in Cluster 2. It was shown that Cluster 4 included the group of six soybean genotypes with the largest seed size, shape, weight, and superior donors (JS 20-30, DS 9712, JS 93-06, PK 564, JS 9423, and UPSL-332). In contrast, Cluster 2 consisted of a group of 26 soybean genotypes with smaller seed size, shape, and weight traits. Tukey’s HSD test was used to select the best contrast donor genotype from a group of 26 genotypes clustered in Cluster 2 (**Supplementary Table 8**). Moreover, a moderate to high positive (0.38 to 0.60) relationship was found between AS and SW traits estimated within the cluster groups. Each of the six selected superior (JS 20-30, DS 9712, JS 93-06, PK 564, JS 9423, and UPSL-332) and contrasting soybean genotypes (BJJF-8, UPSL-365B, M-53, L-768, UPSL-736, UPSL-326, and L-780) for seed size, shape, and hundred-seed weight has been utilized in soybean breeding programs and crop improvement.

**Table 3:** Confusion matrix for validating the list of cluster members (genotypes) identified by CL and AL hierarchical clustering analysis.

Cluster member list	ALHCA			
CLHCA	Cluster 1	Cluster 2	Cluster 3	Cluster 4
Cluster 1	66	0	0	0
Cluster 2	0	26	0	0
Cluster 3	0	0	49	17
Cluster 4	0	0	0	6
Cluster range_AS	15.50-18.30	13.60-15.43	17.98-20.78	22.26-23.80
Cluster range_SW	5.10-7.34	5.10-6.12	6.55-8.16	8.48-9.46
Cluster mean_AS	17.01	14.65	19.41	22.79
Cluster mean_SW	6.33	5.70	7.26	8.85

## Conclusions

An image processing technique was used to estimate the seed architectural traits and hundred-seed weight of 164 different soybean accessions using a flatbed scanner. The image processing software, SmartGrain, was used to obtain their size, shape, and other seed weight-related features. Precision phenotyping guides breeders in the use of genotyping data for crop improvement. Therefore, breeders require defined information from a large set of accessions or germplasm collections for trait improvement. At present, phenotyping is aided by imaging and machine learning techniques in crop plants, as these can screen more accessions in less time and space for our trait of interest. In the current study, image processing technology was used to extract morphological traits such as surface area, length, width, and perimeter from seeds of soybean accessions to predict the final hundred-seed weight. The strength of the correlation between traits revealed that surface area, perimeter length, seed length, and width were good predictors of hundred-seed weight. Machine learning models were successfully implemented to estimate hundred-seed weight based on superior image-based architectural traits. The highest R^2^ value with lower bias and error values was obtained with a multivariate regression model in the testing data set. The donor soybean genotype identified with superior seed size, shape, and weight traits can be utilized for soybean crop improvement programs. The same methodology can be used to breed better crop varieties for higher productivity in other plants.

## Data availability statement

The original contributions presented in the study are included in the article/**Supplementary Material**. Further inquiries can be directed to the corresponding author.

## Author contributions

ND and DR conceived this study. ND and AyR performed the experiment with assistance from DR and AmR. ND and DR implemented the code. DR and AmR worked on data visualization. ND and AyR participated in the analysis with the help of DR and AmR. AyR, AmR, and DR writing—original draft preparation. DR, AmR, SL, RS, and VC writing—review and editing. SL and DR - project administration. VC and RS - supervision. All authors contributed to the article and approved the submitted version.
