# Supplementary material for: Image-based phenotyping of seed architectural traits and prediction of seed weight using machine learning models in soybean
Source: Front Plant Sci. 2023 Sep 12;14:1206357. doi: 10.3389/fpls.2023.1206357 (PMC10523016; doi:10.3389/fpls.2023.1206357)
Supplement: Supplementary file 1 [file DataSheet_1.pdf]

Supplementary figures

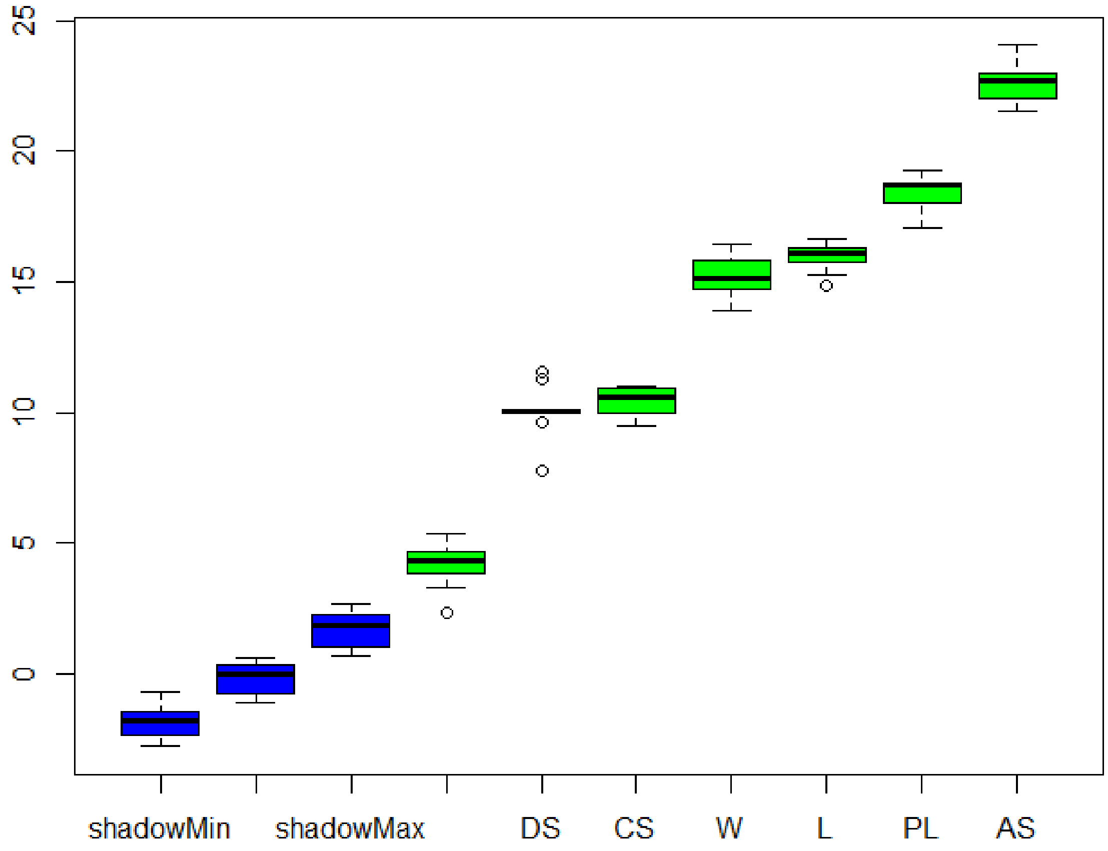

Supplementary figure 1. Boruta Result plot of dependent and independent variables studied.

Training data set **Testing data set**

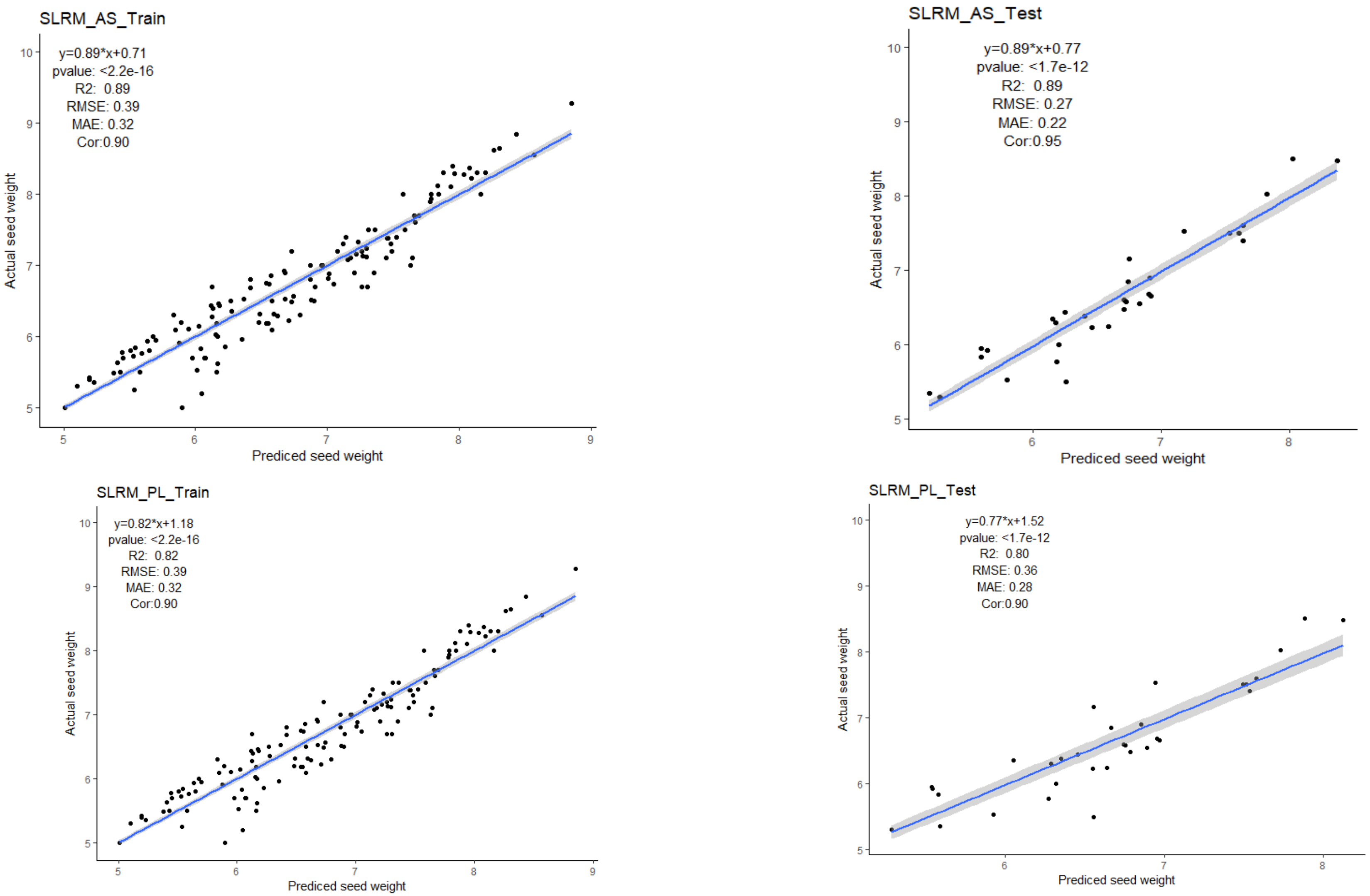

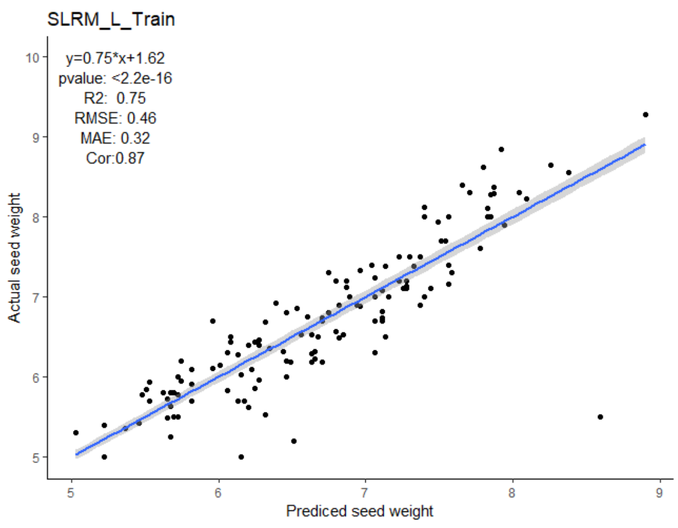
SLRM_L_Train
y=0.75*x+1.62
pvalue: <2.2e-16
R2: 0.75
RMSE: 0.46
MAE: 0.32
Cor:0.87
Actual seed weight
Prediced seed weight
5
6
7
8
9
10

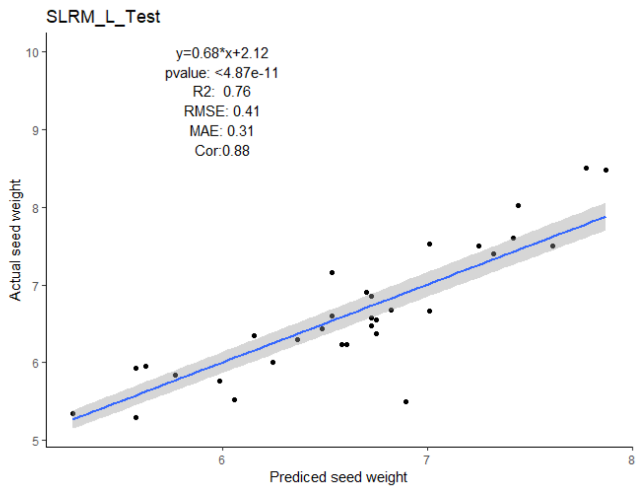
SLRM_L_Test
y=0.68*x+2.12
pvalue: <4.87e-11
R2: 0.76
RMSE: 0.41
MAE: 0.31
Cor:0.88
Actual seed weight
Prediced seed weight
5
6
7
8
9
10

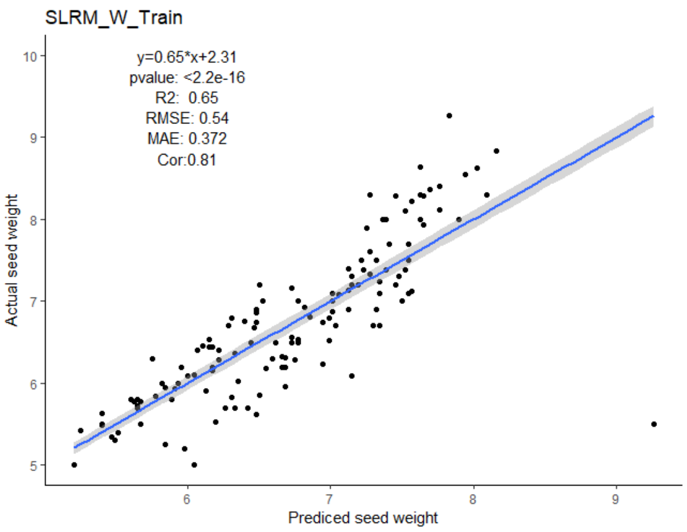
SLRM_W_Train
y=0.65*x+2.31
pvalue: <2.2e-16
R2: 0.65
RMSE: 0.54
MAE: 0.372
Cor:0.81
Actual seed weight
Prediced seed weight
5
6
7
8
9
10

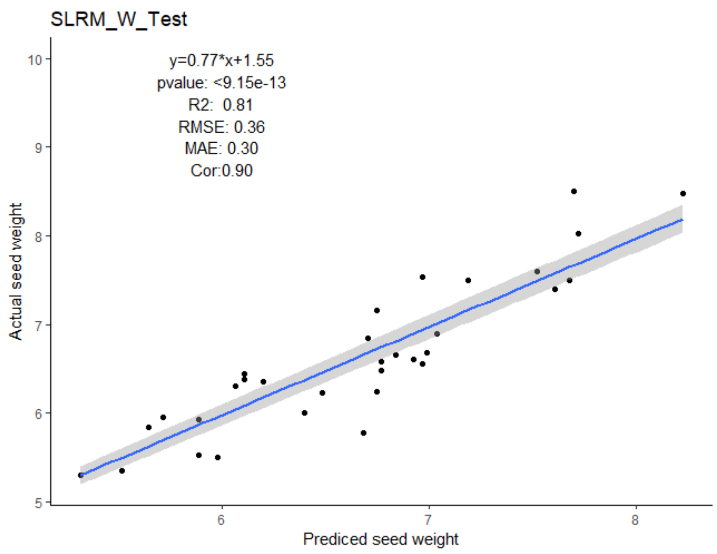
SLRM_W_Test
y=0.77*x+1.55
pvalue: <9.15e-13
R2: 0.81
RMSE: 0.36
MAE: 0.30
Cor:0.90
Actual seed weight
Prediced seed weight
5
6
7
8
9
10

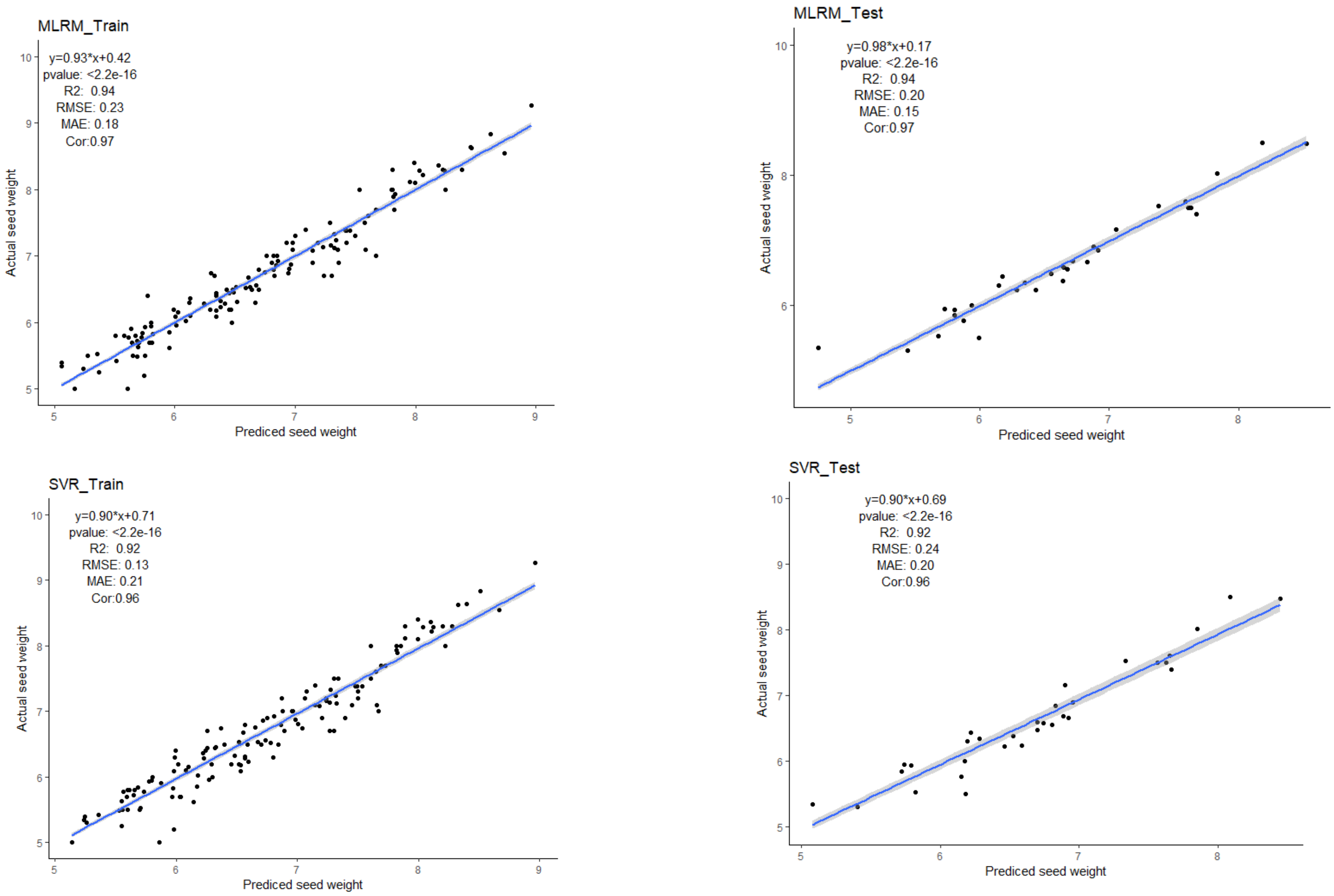
MLRM_Train
y=0.93*x+0.42
pvalue: <2.2e-16
R2: 0.94
RMSE: 0.23
MAE: 0.18
Cor:0.97
Actual seed weight
Prediced seed weight
MLRM_Test
y=0.98*x+0.17
pvalue: <2.2e-16
R2: 0.94
RMSE: 0.20
MAE: 0.15
Cor:0.97
Actual seed weight
Prediced seed weight
SVR_Train
y=0.90*x+0.71
pvalue: <2.2e-16
R2: 0.92
RMSE: 0.13
MAE: 0.21
Cor:0.96
Actual seed weight
Prediced seed weight
SVR_Test
y=0.90*x+0.69
pvalue: <2.2e-16
R2: 0.92
RMSE: 0.24
MAE: 0.20
Cor:0.96
Actual seed weight
Prediced seed weight

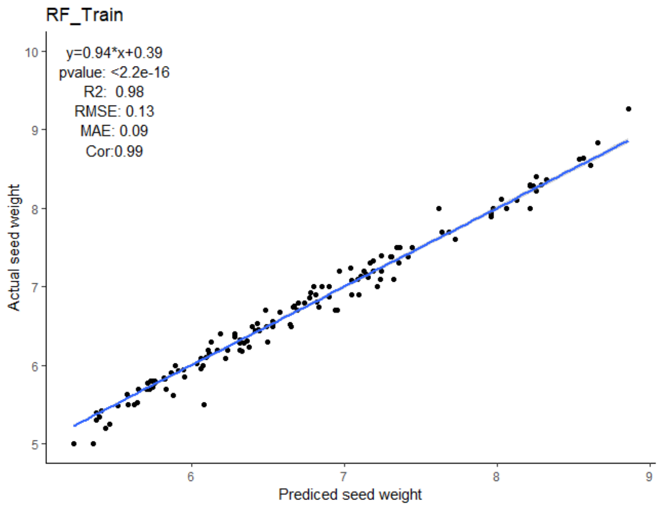
RF_Train
y=0.94*x+0.39
pvalue: <2.2e-16
R2: 0.98
RMSE: 0.13
MAE: 0.09
Cor:0.99
Actual seed weight
Prediced seed weight

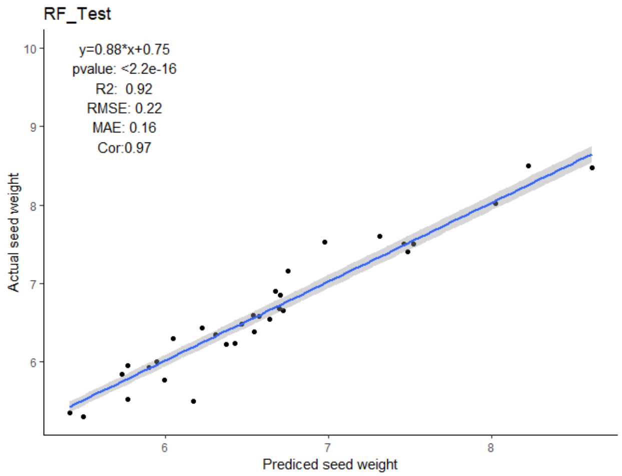
RF_Test
y=0.88*x+0.75
pvalue: <2.2e-16
R2: 0.92
RMSE: 0.22
MAE: 0.16
Cor:0.97
Actual seed weight
Prediced seed weight

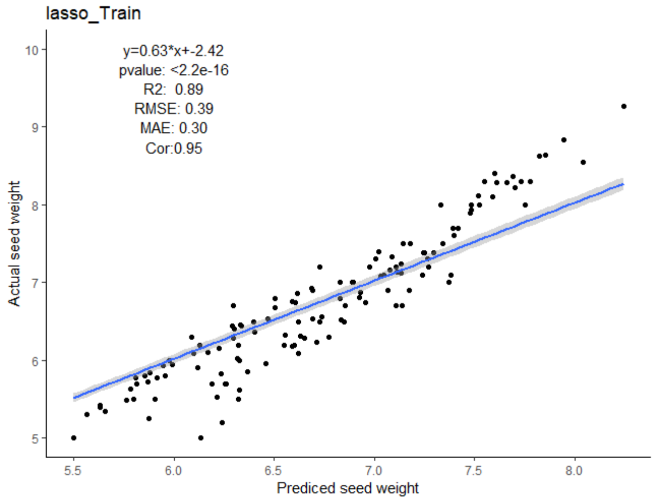
lasso_Train
y=0.63*x+-2.42
pvalue: <2.2e-16
R2: 0.89
RMSE: 0.39
MAE: 0.30
Cor:0.95
Actual seed weight
Prediced seed weight

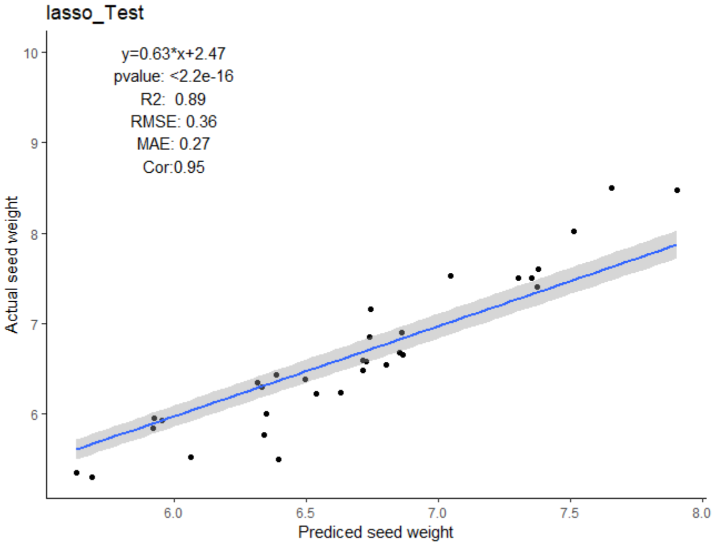
lasso_Test
y=0.63*x+2.47
pvalue: <2.2e-16
R2: 0.89
RMSE: 0.36
MAE: 0.27
Cor:0.95
Actual seed weight
Prediced seed weight

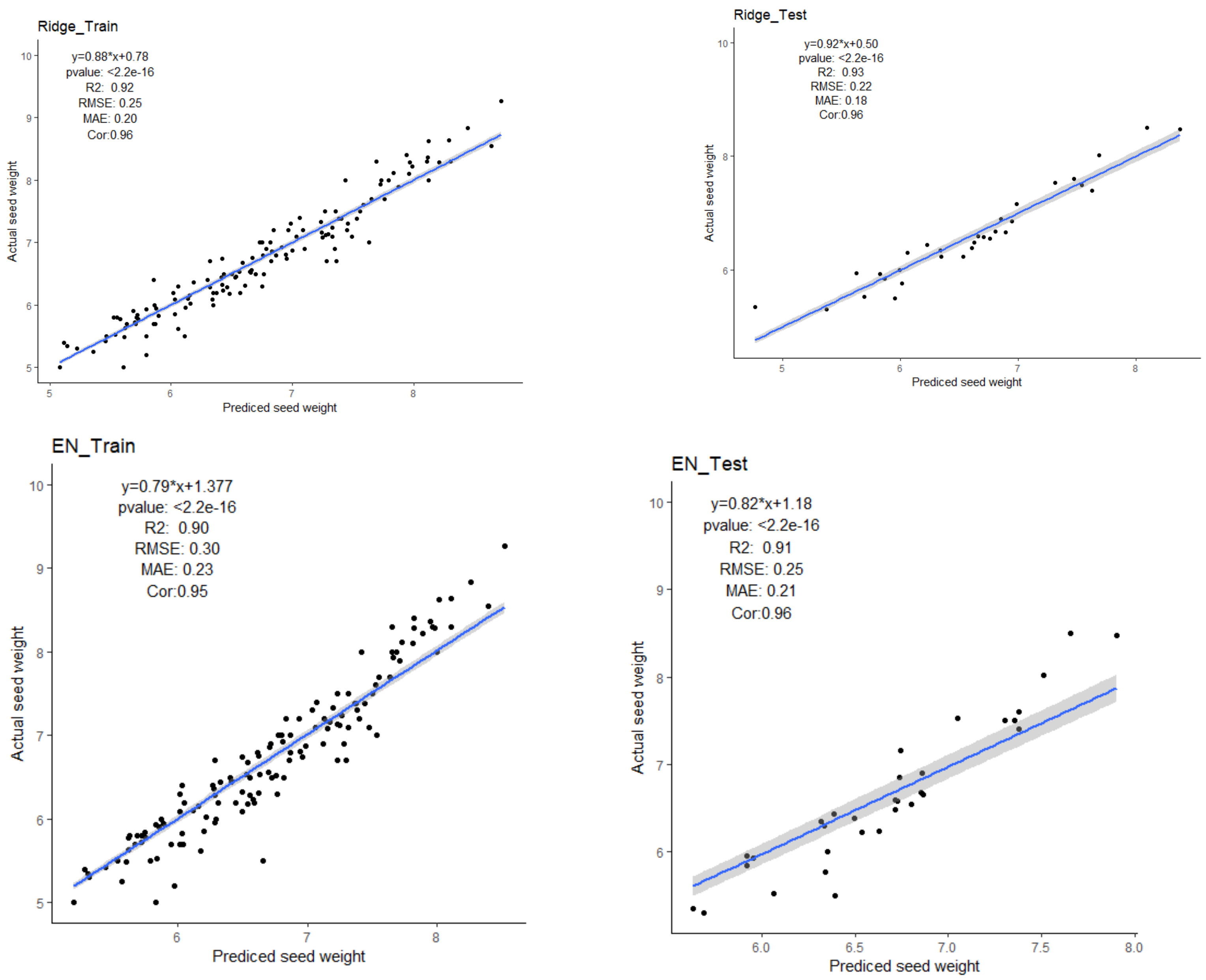

Ridge_Train
y=0.88*x+0.78
pvalue: <2.2e-16
R2: 0.92
RMSE: 0.25
MAE: 0.20
Cor:0.96
Actual seed weight
Prediced seed weight
Ridge_Test
y=0.92*x+0.50
pvalue: <2.2e-16
R2: 0.93
RMSE: 0.22
MAE: 0.18
Cor:0.96
Actual seed weight
Prediced seed weight
EN_Train
y=0.79*x+1.377
pvalue: <2.2e-16
R2: 0.90
RMSE: 0.30
MAE: 0.23
Cor:0.95
Actual seed weight
Prediced seed weight
EN_Test
y=0.82*x+1.18
pvalue: <2.2e-16
R2: 0.91
RMSE: 0.25
MAE: 0.21
Cor:0.96
Actual seed weight
Prediced seed weight

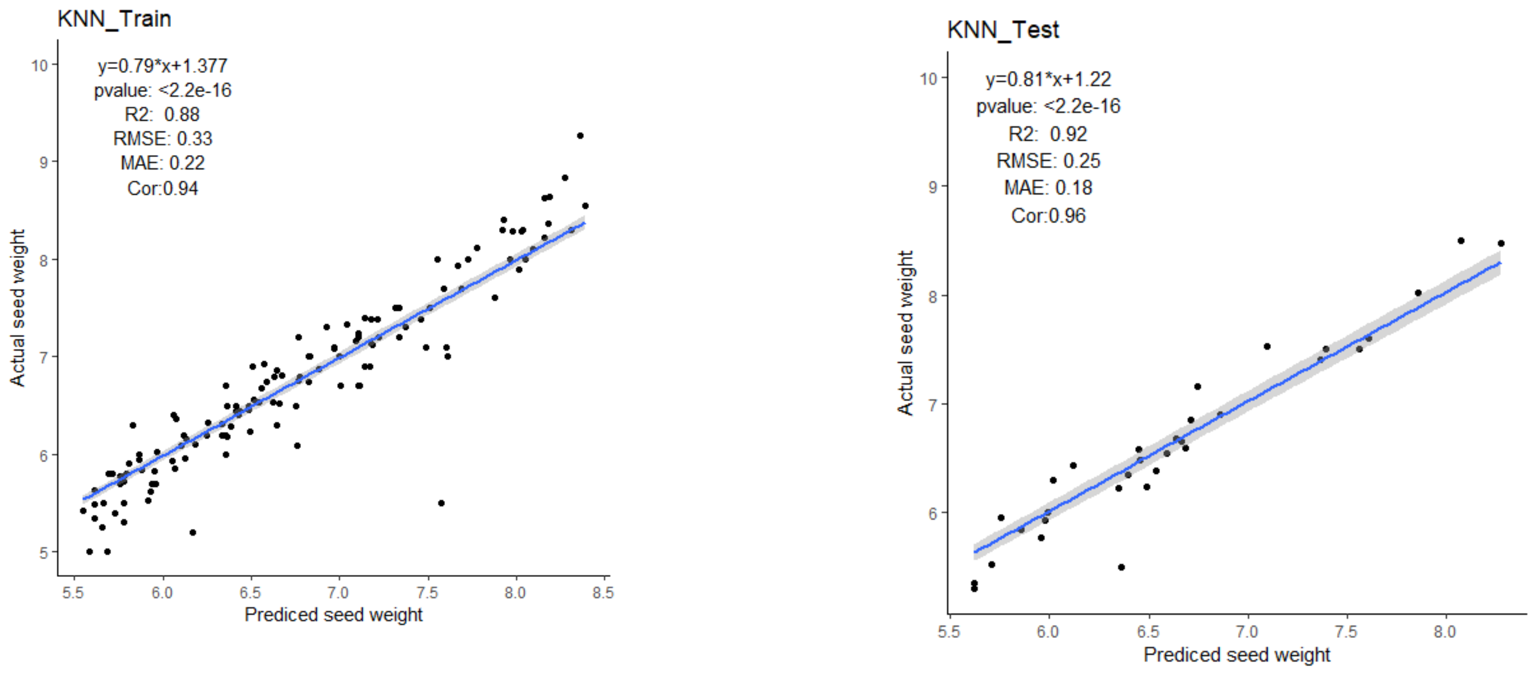

Supplementary figure 2. Boruta result plots of dependent and independent variables studied.

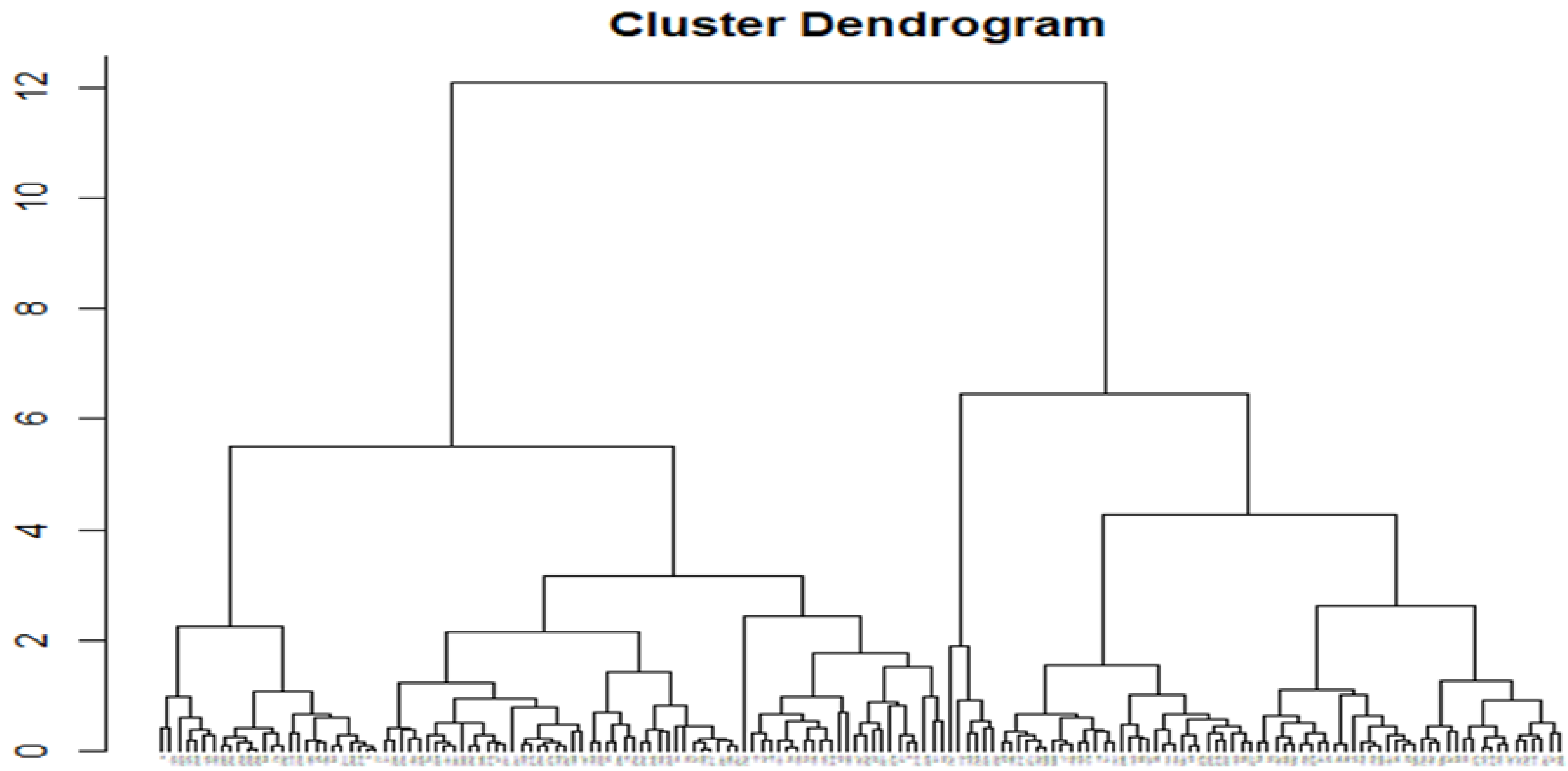

Supplementary figure 3A: Hierarchical clustering analysis of 164 diverse soybean genotypes using complete linkage method.

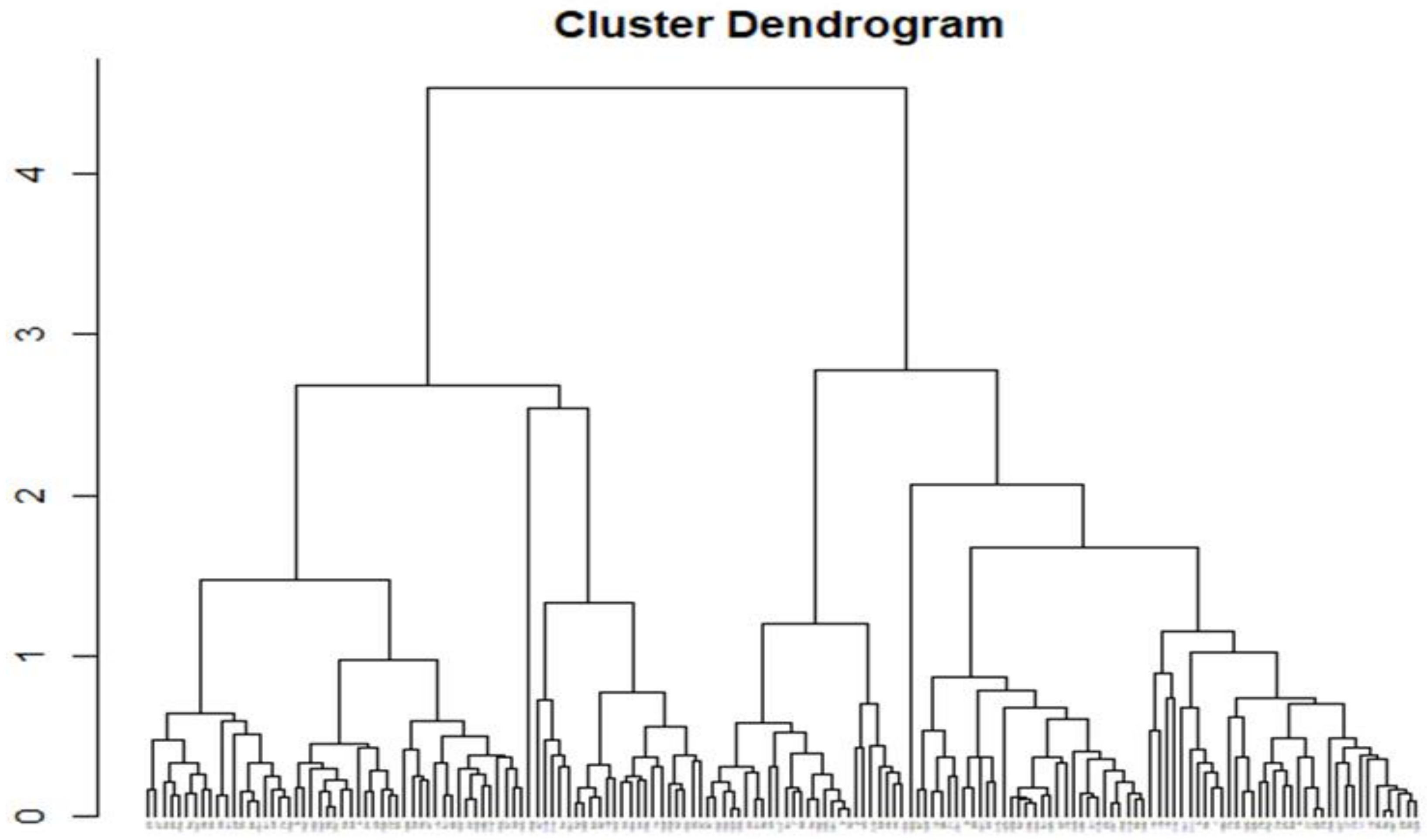

Supplementary figure 3B: Hierarchical clustering analysis of 164 diverse soybean genotypes using average linkage method.
